# Hyperpolarization electrical signals induced by local action of moderate heating influence photosynthetic light reactions in wheat plants

**DOI:** 10.3389/fpls.2023.1153731

**Published:** 2023-04-05

**Authors:** Lyubov Yudina, Ekaterina Sukhova, Alyona Popova, Yuriy Zolin, Karina Abasheva, Kseniya Grebneva, Vladimir Sukhov

**Affiliations:** Department of Biophysics, N.I. Lobachevsky State University of Nizhny Novgorod, Nizhny Novgorod, Russia

**Keywords:** photosynthetic response, quantum yield of photosystem II, non-photochemical quenching, local moderate heating, soil drought, hyperpolarization electrical signals, light

## Abstract

Local action of stressors induces fast changes in physiological processes in intact parts of plants including photosynthetic inactivation. This response is mediated by generation and propagation of depolarization electrical signals (action potentials and variation potentials) and participates in increasing plant tolerance to action of adverse factors. Earlier, we showed that a local action of physiological stimuli (moderate heating and blue light), which can be observed under environmental conditions, induces hyperpolarization electrical signals (system potentials) in wheat plants. It potentially means that these signals can play a key role in induction of fast physiological changes under the local action of environmental stressors. The current work was devoted to investigation of influence of hyperpolarization electrical signals induced by the local action of the moderate heating and blue light on parameters of photosynthetic light reactions. A quantum yield of photosystem II (Ф_PSII_) and a non-photochemical quenching of chlorophyll fluorescence (NPQ) in wheat plants were investigated. It was shown that combination of the moderate heating (40°C) and blue light (540 µmol m^-2^s^-1^) decreased Ф_PSII_ and increased NPQ; these changes were observed in 3-5 cm from border of the irritated zone and dependent on intensity of actinic light. The moderate soil drought (7 days) increased magnitude of photosynthetic changes and shifted their localization which were observed on 5-7 cm from the irritated zone; in contrast, the strong soil drought (14 days) suppressed these changes. The local moderate heating decreased Ф_PSII_ and increased NPQ without action of the blue light; in contrast, the local blue light action without heating weakly influenced these parameters. It meant that just local heating was mechanism of induction of the photosynthetic changes. Finally, propagation of hyperpolarization electrical signals (system potentials) was necessary for decreasing Ф_PSII_ and increasing NPQ. Thus, our results show that hyperpolarization electrical signals induced by the local action of the moderate heating inactivates photosynthetic light reactions; this response is similar with photosynthetic changes induced by depolarization electrical signals. The soil drought and actinic light intensity can influence parameters of these photosynthetic changes.

## 1 Introduction

Local action of stressors can induce systemic adaptation responses based on generation and propagation of various long-distance stress signals. It is known that electrical signals (ESs) are an important type of the long-distance signals (Choi et al., 2016; Choi et al., 2017; Sukhova and Sukhov, 2021) which induce fast changes in plant physiological processes (Fromm and Lautner, 2007; van Bel et al., 2014; Gallé et al., 2015; Hedrich et al., 2016; Szechyńska-Hebda et al., 2017, Szechyńska-Hebda et al., 2022; Farmer et al., 2020). ESs include two depolarization electrical signals (DESs), namely, action potential and variation potential, and one hyperpolarization electrical signal (HES), namely, system potential (Szechyńska-Hebda et al., 2017; Sukhova and Sukhov, 2021). DES includes initial depolarization and following repolarization; HES includes hyperpolarization and following repolarization.

Action potential (Trebacz et al., 2006; Felle and Zimmermann, 2007; Sukhov et al., 2019) is a short-term self-propagating spike which can be caused by not involving tissue damage stimuli and has a long-term refractory period. Generation of action potential is initiated by activation of potential-dependent Ca^2+^ channels in the plasma membrane and calcium influx which activates anion channels and inactivate H^+^-ATPase in the plasma membrane (Fromm and Lautner, 2007; Sukhova and Sukhov, 2021). Variation potential is a long-term DES with irregular shape (Stahlberg et al., 2006; Fromm and Lautner, 2007; Sukhova and Sukhov, 2021) which can be caused by local damages (mainly, local burning and heating to high temperatures). Variation potential is considered as a local electrical response to propagation of non-electrical signals (Sukhova and Sukhov, 2021) which can be chemical signals (Fromm and Lautner, 2007; Toyota et al., 2018), hydraulic signals (Stahlberg and Cosgrove, 1997; Mancuso, 1999), or their combinations (Malone, 1994). It is hypothesized that these signals activate ligand-dependent or mechano-sensitive Ca^2+^ channels in the plasma membrane and, thereby, induce Ca^2+^ influx; strong increasing Ca^2+^ concentration in the cytoplasm induces transient inactivation of H^+^-ATPase and, probably, changes in activity of ion channels in the plasma membrane (Stahlberg and Cosgrove, 1997; Sukhova and Sukhov, 2021). System potential is a long-term HES which can be induced by various irritations (including damages, Lautner et al., 2005; Yudina et al., 2022) and is based on a transient activation of H^+^-ATPase (Zimmermann et al., 2009; Zimmermann et al., 2016). Earlier, we hypothesized that system potential can be related to propagation of the hydraulic signals with low amplitude, moderate activation of mechanosensitive Ca^2+^ channels in the plasma membrane, calcium influx and relatively low increase of the calcium concentration in the cytoplasm which induces the transient activation of H^+^-ATPase (Yudina et al., 2022; Yudina et al., 2023).

Induction and propagation of DESs can strongly regulate physiological processes in plants (Fromm and Lautner, 2007; Gallé et al., 2015; Sukhova and Sukhov, 2021). They influence expression of defense genes (Wildon et al., 1992; Mousavi et al., 2013), synthesis of phytohormones (Dziubinska et al., 2003; Hlavácková et al., 2006; Hlavinka et al., 2012; Krausko et al., 2017), photosynthetic processes (Fromm and Lautner, 2007; Gallé et al., 2015; Szechyńska-Hebda et al., 2017; Sukhova and Sukhov, 2021), transport of sugars in phloem (Fromm and Bauer, 1994; Furch et al., 2009; Furch et al., 2010), respiration (Filek and Kościelniak, 1997; Pavlovič et al., 2011; Lautner et al., 2014), transpiration (Kaiser and Grams, 2006; Grams et al., 2007; Vuralhan-Eckert et al., 2018), growth (Shiina and Tazawa, 1986; Stahlberg and Cosgrove, 1996), and others. It is interesting that influence of action potential and variation potential on physiological processes is often similar (Sukhov et al., 2019) supporting universal mechanisms of DESs-induced physiological changes in plants.

Mechanisms of influence of DESs on physiological processes are discussed (Sukhov, 2016; Sukhova and Sukhov, 2021); however, an important mechanism of physiological changes is the transient inactivation of H^+^-ATPase in the plasma membrane causing alkalization of the apoplast and acidification of the cytoplasm, stroma and lumen of chloroplast. These pH changes participate in DESs-induced changes in photosynthetic processes (through decrease of a mesophyll CO_2_ conductance and suppression of photosynthetic dark and light reactions, Pavlovič et al., 2011; Gallé et al., 2013) and respiration (Sukhova and Sukhov, 2021). Other mechanisms are also considered; e.g., direct influence of the DESs-induced increase of the Ca^2+^ concentration (Bulychev and Alova, 2022) or reactive oxygen species (Białasek et al., 2017) on photosynthesis.

A result of DESs-induced changes in physiological processes can be increasing plant tolerance to action of stressors (Choi et al., 2017; Sukhova and Sukhov, 2021). This hypothesis is supported by the increased tolerance of biological membranes and photosynthetic machinery after induction and propagation of electrical signals (Retivin et al., 1997; Retivin et al., 1999; Szechyńska-Hebda et al., 2010; Sukhov, 2016; Zandalinas et al., 2020a; Zandalinas et al., 2020b). DESs-induced activation of photosynthetic defense mechanisms (e.g., activation of a non-photochemical quenching of chlorophyll fluorescence, NPQ), increase of ATP content stimulating reparation processes, decrease of transpiration supporting water content in plants, and modification of processes of programmed cell death are considered as potential mechanisms of the increased plant tolerance to stressors (Sukhov, 2016; Sukhova and Sukhov, 2021). Relations between electrical activity and plant tolerance are theoretical basis of methods of estimation of plant changes under action of stressors, which are actively developed in numerous works (Retivin et al., 1997; Chatterjee et al., 2015; Saraiva et al., 2017; Chatterjee et al., 2018; Simmi et al., 2020; Parise et al., 2021).

However, the hypothesis about a key role of DESs in induction of the fast systemic response to the local action of stressors and increase of the plant tolerance to adverse factors has some problems. In our earlier review (Sukhov et al., 2019), we summarized arguments showing that action potential, which can be induced by irritations with weak and moderate intensity (Retivin et al., 1997), should not be often observed in higher plants under environmental conditions because long-term rest interval and absence of chronic action of stressors are necessary for propagation of this DES. In contrast, variation potential can propagate under chronic action of stressor (e.g., under moderate water deficit, Yudina et al., 2022); however, the induction of this DES requires strong local damages (mainly, burning or heating to about 55°C and more, Sukhova and Sukhov, 2021). Action of these damages can be observed under extreme situations (e.g., wildfire); it means that the propagation of variation potential cannot be a common phenomenon under environmental conditions, either.

In our previous work (Yudina et al., 2023), we investigated possibility of induction of variation potential under combined local action of moderate heating (40°C) and blue light because this combination can often influence plants under environmental conditions and its action can be local. Our results did not show propagation of variation potentials; however, combined action of the moderate heating and blue light (or the moderate heating only) induced the propagation of HESs, which were classified as system potential. Propagation of HESs was also observed under the moderate soil drought; however, the strong soil drought prevented this propagation. These results show that hyperpolarization signal can play important role in induction of the plant systemic response to local action of stressors under environmental conditions. Checking this hypothesis requires to answer on the following questions: Can these HESs influence physiological processes in plants? and Is this influence similar to the well-investigated influence of DESs?

Thus, the general aim of our work was investigation of influence of HES-inducing irritations on parameters of photosynthetic light reactions including NPQ and effective quantum yield of photosystem II (Ф_PSII_), which are key parameters of photosynthetic light reactions and show efficiency of the linear electron flow through the electron transport chain of chloroplasts and stress changes in photosynthetic machinery, respectively (Maxwell and Johnson, 2000; Ruban, 2016). It is known that both parameters are an important target of influence of DESs inducing decrease of Ф_PSII_ and increase of NPQ (Sukhov, 2016); their DESs-induced changes increase the plant tolerance to action of stressors.

To solve this problem, we approximately repeated design of experiments from our previous work (Yudina et al., 2023); however, the current work was focused on influence of the local irritations on NPQ and Ф_PSII_; electrical signals were measured in separate experimental series only. The following specific questions were analyzed:

- Could combination of the local action of the blue light and moderate heating induce changes in NPQ and Ф_PSII_ in irrigated wheat plants? Detection of these changes was basis of further analysis of the possible HESs influence on photosynthesis.
- Could this combination induce changes in NPQ and Ф_PSII_ in wheat plants under the moderate and strong soil drought? If HESs participated in photosynthetic regulation, it could be expected that these changes should be large under the moderate drought and low under the strong drought.
- Could the local action of the blue light only or the local action of the moderate heating only induce changes in NPQ and Ф_PSII_ in wheat plants? It was previously shown (Yudina et al., 2023) that the moderate heating induced HESs, which were similar to HESs caused by the local action of combination of the blue light and moderate heating; in contrast, the blue light induced HESs with decreased amplitudes. It meant that the moderate heating played a key role in the induction of HESs. If HESs participated in the photosynthetic regulation, it could be expected that the local moderate heating should induce large changes in NPQ and Ф_PSII_; in contrast, the local action of the blue light should cause weak changes in these parameters.
- Could properties of HESs measured in the current work be approximately similar to these properties showed in our previous work (Yudina et al., 2023)? Response on this question should additionally support/disprove the HESs participation in the photosynthetic regulation.

## 2 Materials and methods

### 2.1 Plant material and soil drought

Spring wheat plants (*Triticum aestivum* L., cultivar “Daria”) were used in the current investigation in accordance with Yudina et al. (2023) because this cultivar is characterized by moderate tolerance to drought and is suitable for investigation of the soil drought influence on photosynthetic responses. Wheat seeds were provided by Federal Research Center N. I. Vavilov All-Russian Institute of Plant Genetic Resources (VIR) (St.Petersburg, Russia).

Plants were cultivated in the vegetation room of Department of Biophysics (N.I. Lobachevsky State University of Nizhny Novgorod, Nizhny Novgorod, Russia). Duration of soaking was 2 days; after that, wheat seedlings were planted into pots with the universal soil. 16 h photoperiod and 24°C temperature were used for the wheat cultivation. Light source was luminescent lamps FSL YZ18RR (Foshan Electrical And Lighting Co., Ltd, Foshan, China); light intensity was about 100 µmol m^-2^s^-1^. 13-14 days old wheat plants were used for photosynthetic and electrophysiological measurements in the most of experiments excluding investigation of influence of the soil drought.

In the case of the soil drought, 13-14 days old wheat plants were divided into control group (with periodical irrigation every 2 days) and experimental group (without this irrigation). Photosynthetic and electrophysiological measurements were performed in 7 days (the moderate soil drought) and 14 days (the strong soil drought) after termination of irrigation. As a result, these measurements were carried out on 20-21 days old plants and 27-28 days old plants in investigation of influence of the moderate and strong soil drought, respectively. The relative water content (RWC) was measured in wheat shoots as $\frac{\text{FW}-\text{DW}}{\text{FW}}100\%$ , where FW is the fresh weight and DW is the dry weight (Jin et al., 2017). DW was measured after 2 h of 100°C temperature action in a TV-20-PZ-K thermostat (Kasimov Instrument Plant, Kasimov, Russia). It was shown that RWCs were 91.8 ± 0.3% (control) and 90.9 ± 0.1% (experiment) under the 7-day drought and 90.7 ± 0.2% (control) and 73.7 ± 5.0% (experiment) under the 14-day drought (*n*=6-12); differences between control and experimental wheat plants were significant.

Photosynthetic measurements (see Section 2.3 for details) showed that the soil drought decreased Ф_PSII_ and NPQ. The effective quantum yield of photosystem II was 0.504 ± 0.004 (irrigation), 0.459 ± 0.006 (the 7-day soil drought), and 0.200 ± 0.012 (the 14-day soil drought). The non-photochemical quenching was 0.903 ± 0.019 (irrigation), 1.213 ± 0.058 (the 7-day soil drought), and 2.128 ± 0.326 (the 14-day soil drought). Differences between all values were significant.

These result showed that the soil drought decreased RWC, disrupted photosynthetic processes, and, therefore, could be used in the further investigation.

### 2.2 Plant irritations

**Figure 1:**
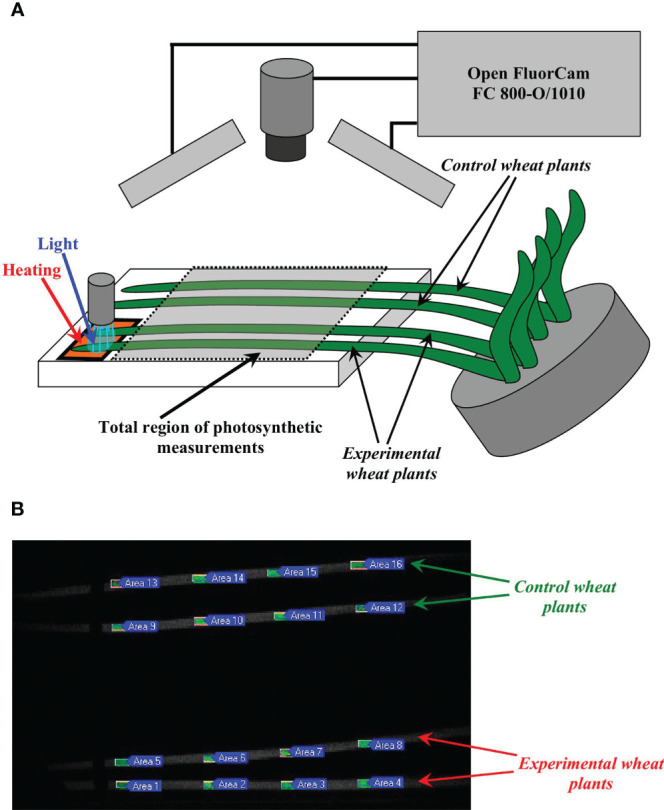
Scheme of experimental set providing to local action of heating and blue light on top of wheat plants and to photosynthetic measurements **(A)**, and example of localizations of ROIs in experimental and control wheat plants **(B)**. Local action of heating (40°C) and blue light (540 µmol m^-2^s^-1^) were described in detail in our previous work (Yudina et al., 2023); duration of illumination equaled to 10 min after initiation of irritation, and duration of heating equaled to about 45 min. PAM imaging Open FluorCam FC 800-O/1010 was used for measurements of quantum yield of photosystem II (Ф_PSII_) and non-photochemical quenching of chlorophylls (NPQ). Photosynthetic measurements were initiated in 15 min before the irritation after 15-min dark adaptation and subsequent 30-min adaptation under standard white actinic light of this system (86, 456, or 838 µmol m^-2^s^-1^). Four wheat plants (two plants with irritations and two control plants) were simultaneously measured in each experiment. In each plant, Ф_PSII_ and NPQ were investigated in four ROIs with centers in 3, 5, 7, and 9 cm from the border of the irritated zone.

Plant irritations were described in our previous work (Yudina et al., 2023) in detail. Briefly, the combination of blue light (540 µmol m^-2^s^-1^) and heating (40°C) was used as the main irritation (**Figure 1A**); the blue light only or the heating only were used in separate series of experiments. Total size of the irritated zone was about 4 cm from the top of the second mature leaf. Duration of the blue light action was 10 min after initiation of this irritation; duration of the heating was about 45 min (until termination of experiment). In experiments with photosynthetic measurements, four wheat plants were simultaneously investigated in each repetition; two plants were irritated (experiment) and two plants were not irritated (control). In experiments with electrophysiological measurements, one plant was used in each repetition. All wheat plants were fixed before experiments.

A self-manufactured system with regulated light intensity and heating was used for irritation. A system included blue light LED TDS-P003L4C04, 460 nm, 40 lm, 3 W (TDS Lighting Co., Huishan district, Wuxi city, Jiangsu Province, China) and the Peltier element STORM-71, 3.6 A, 36 W (Kryotherm, St.Petersburg). It was equipped by black tube to prevent illumination of other parts of plants. A light flux meter PM100D with sensor S120C (Thorlabs Ultrafast Optoelectronics, Ann Arbor, Michigan, United States) was used for measurement of light intensity. A thermometer monitor ATE-9380 (Aktakom, Moscow, Russia) was used for measurement of the leaf temperature.

### 2.3 Measurements of Ф_PSII_, NPQ, and leaf temperature

A PAM imaging Open FluorCam FC 800-O/1010 (Photon Systems Instruments, Drasov, Czech Republic) was used for measuring Ф_PSII_ and NPQ in wheat plants (**Figure 1A**). The maximum fluorescence (Fm) (Maxwell and Johnson, 2000) was measured after the 15-min dark adaptation with using the standard saturation pulse of this system (SP, 4000 μmol m^-2^ s^-1^, cold white light, 6500 K). After subsequent 30-min adaptation under a white actinic light provided by the system, saturation pulses were generated every 90 s under this actinic light to measure the maximum fluorescence in the light (Fm’) and steady-state value of fluorescence immediately prior to the saturation pulse (Ft) (Maxwell and Johnson, 2000). 456 µmol m^-2^s^-1^ intensity of the actinic light was used in the most of experiments; the 86 and 838 µmol m^-2^s^-1^ intensities were used as additional intensities to investigate influence of the actinic light intensity on local irritation-induced photosynthetic changes. Ф_PSII_ and NPQ were calculated on basis of Fm, Fm’, and F in accordance with standard equations (Maxwell and Johnson, 2000).

Local irritations were initiated after 15-min measurement of photosynthetic parameters; control plants were not irritated. After that, Ф_PSII_ and NPQ were measured for 45 min. We analyzed photosynthetic parameters in four ROIs with same length (about 0.5 cm) in each wheat plant (the second mature leaf) with centers located on 3, 5, 7, and 9 cm from the irritated zone (**Figure 1B**).

In the analysis, we used ΔФ_PSII_ and ΔNPQ, which were calculated as Ф_PSII_ – Ф_PSII_^0^ and NPQ – NPQ^0^, respectively. Ф_PSII_^0^ and NPQ^0^ were Ф_PSII_ and NPQ before irritation, which were calculated as averaged Ф_PSII_ and NPQ for three time points (for 10.5–13.5 min from initiation of the photosynthetic measurements). Using ΔФ_PSII_ and ΔNPQ decreased individual variability of photosynthetic parameters in investigated wheat plants.

Considering possible influence of the actinic light intensity on a leaf temperature (t_leaf_), we measured t_leaf_ in 3 cm from the irritated zone before initiation of action of stressors. The Testo 885 thermal imager (Testo, Lenzkirch, Germany) was used for these measurements.

### 2.4 Measurements of hyperpolarization electrical signals

In accordance with Yudina et al. (2023), a system including extracellular Ag^+^/AgCl electrodes (RUE Gomel Measuring Equipment Plant, Gomel, Belarus), a high-impedance IPL-113 amplifier (Semico, Novosibirsk, Russia), and a personal computer was used for extracellular measurements of HESs. Reference electrode was placed on the wheat stem (near the soil). We only used one measuring electrode placed in 5 cm from the irritated zone to simplify this additional experimental analysis. This distance was used because photosynthetic changes were mainly localized within 5 cm from the irritated zone (see Section 3); i.e., HESs should propagate through this point to induce photosynthetic changes in wheat leaf.

### 2.5 Statistics

Each experimental or control group included from 7 to 24 separate plants; quantities of plants were shown in caption of figures. Representative records, mean values, and standard errors were presented in the figures. Significant differences were determined according to the Student’s t-test.

## 3 Results

### 3.1 Local action of combination of heating and blue light on photosynthetic parameters under irrigated conditions

In the first stage of the current research, influence of local action of combination of the moderate heating and blue light on Ф_PSII_ and NPQ in non-irritated parts of leaf in wheat plants were investigated. Actinic light with 86, 456, and 838 µmol m^-2^s^-1^ intensities was used in these experiments to additionally estimate influence of light conditions on irritation-induced photosynthetic changes.

**Figure 2:**
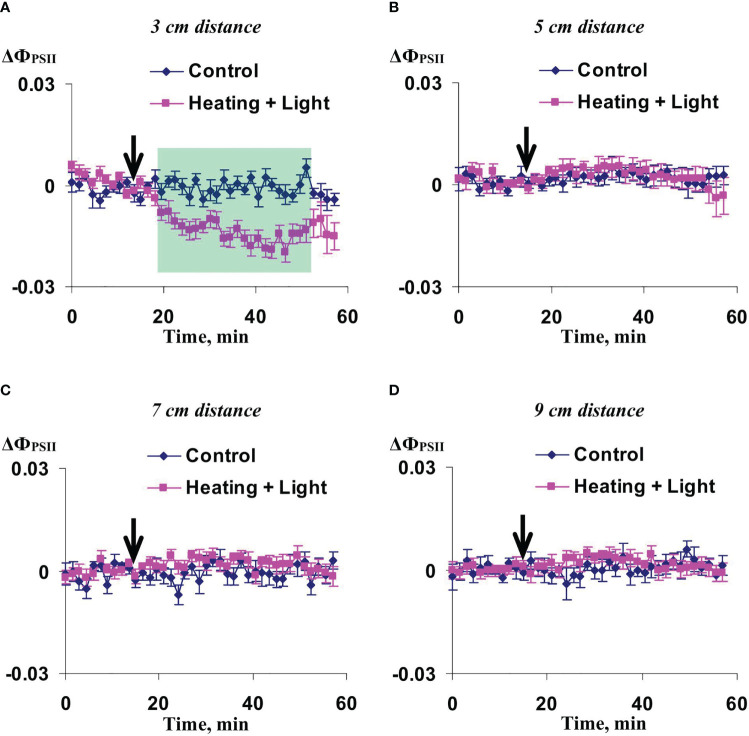
Averaged changes in the quantum yield of photosystem II (ΔФ_PSII_) in 3 cm **(A)**, 5 cm **(B)**, 7 cm **(C)**, and 9 cm **(D)** from zone of the local action of combination of heating and blue light in irrigated wheat plants (*n*=12). Arrow marks initiation of this action; control plants were not irritated. ΔФ_PSII_ was calculated as Ф_PSII_ - Ф_PSII_^0^, where Ф_PSII_^0^ was measured before the initiation of the irritation. Intensity of the white actinic light was 456 µmol m^-2^s^-1^. Green shading shows time interval with significant differences between experimental and control values (*p*<0.05, Student’s test).

Experiments with the 456 µmol m^-2^s^-1^ actinic light intensity showed (**Figure 2A**) that the local action of heating and blue light decreased the quantum yield of photosystem II in 3 cm from the irritated zone. Difference between ΔФ_PSII_ in irritated and control plants reached a maximum value for about 25-30 min after initiation of the local action of heating and blue light; after that, this value decreased. In contrast, significant differences between ΔФ_PSII_ in irritated and control plants were absent at 5 cm (**Figure 2B**), 7 cm (**Figure 2C**), and 9 cm (**Figure 2D**).

**Figure 3:**
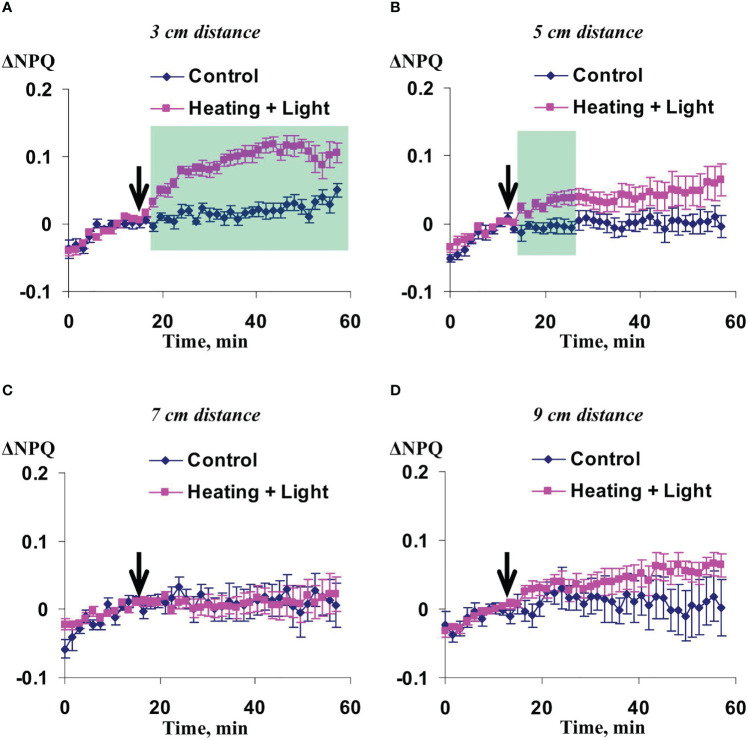
Averaged changes in the non-photochemical quenching of chlorophyll (ΔNPQ) in 3 cm **(A)**, 5 cm **(B)**, 7 cm **(C)**, and 9 cm **(D)** from zone of the local action of combination of heating and blue light in irrigated wheat plants (*n*=12). Arrow marks initiation of this action; control plants were not irritated. ΔNPQ was calculated as NPQ - NPQ^0^, where NPQ^0^ was measured before the initiation of the irritation. Intensity of the white actinic light was 456 µmol m^-2^s^-1^. Green shadings show time intervals with significant differences between experimental and control values (*p*<0.05, Student’s test).

Significant increase of NPQ induced by the local action of heating and blue light was observed in 3 cm (**Figure 3A**) and 5 cm (**Figure 3B**) from the irritated zone. It should be noted that magnitude and duration of this increase in the first point were strongly higher than ones in the second point. The influence of local irritation on NPQ was not significant in 7 cm (**Figure 3C**) and 9 cm (**Figure 3D**).

**Figure 4:**
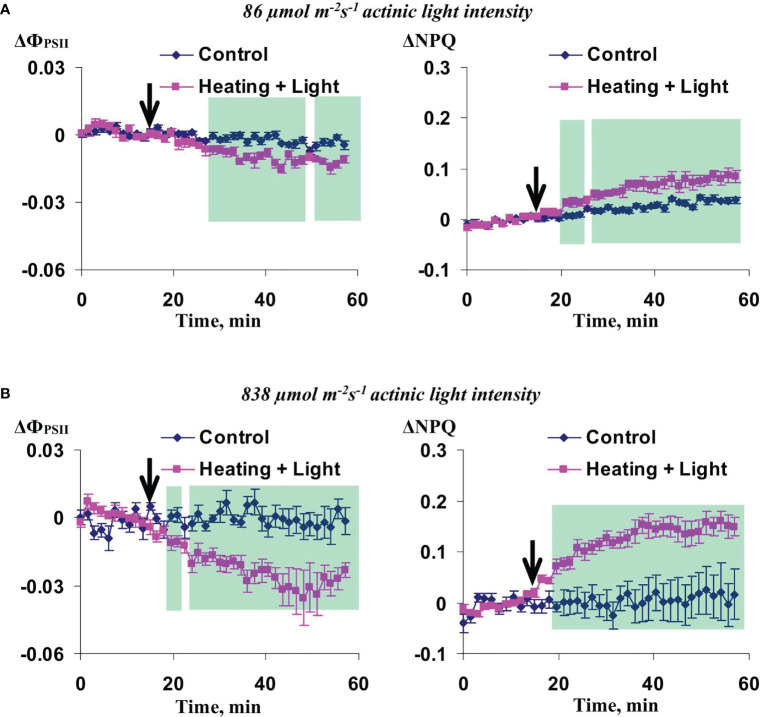
Averaged changes in the quantum yield of photosystem II (ΔФ_PSII_) and non-photochemical quenching of chlorophyll (ΔNPQ) in 3 cm from zone of the local action of combination of heating and blue light in wheat plants under 86 µmol m^-2^s^-1^ **(A)** and 838 µmol m^-2^s^-1^ **(B)** intensity of the white actinic light (*n*=8). Arrow marks initiation of this action; control plants were not irritated. Green shadings show time intervals with significant differences between experimental and control values (*p*<0.05, Student’s test).

Additional analysis of changes in Ф_PSII_ and NPQ induced by the local action of heating and blue light under low and high intensity of the actinic light showed that magnitude of these changes was low under the 86 µmol m^-2^s^-1^ actinic light intensity and high under the 838 µmol m^-2^s^-1^ in 3 cm from the irritated zone (**Figure 4**). With increasing distance from this zone, magnitudes of photosynthetic changes were low and insignificant (data not shown). These results showed that the actinic light intensity influenced magnitude of irritation-induced photosynthetic changes and did not modify dynamics of these changes and their dependence on distance from the irritated zone; as a result, we used the intermediate actinic light intensity (456 µmol m^-2^s^-1^) in the further investigation.

**Figure 5:**
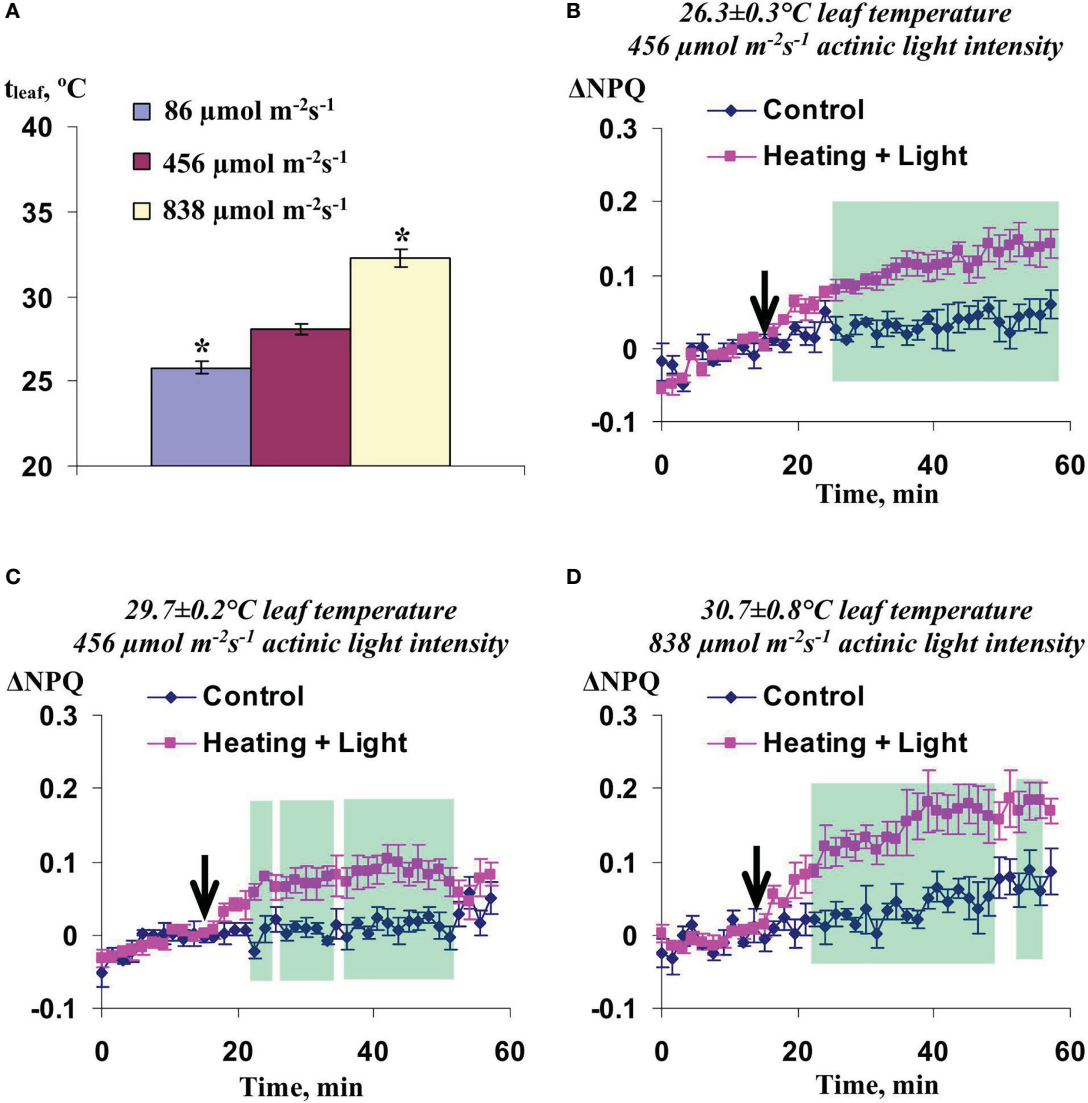
Leaf temperatures (t_leaf_) under different intensities of the actinic light (*n*=16-24) **(A)** and averaged changes in the non-photochemical quenching of chlorophyll (ΔNPQ) in 3 cm from zone of the local action of combination of heating and blue light in wheat plants under the 26.3 ± 0.3°C leaf temperature and 456 µmol m^-2^s^-1^ actinic light intensity (*n*=4) **(B)**, under the 29.7 ± 0.2°C leaf temperature and 456 µmol m^-2^s^-1^ actinic light intensity (*n*=4) **(C)**, and under the 30.7 ± 0.8°C leaf temperature and 838 µmol m^-2^s^-1^ actinic light intensity (*n*=4) **(D)**. Leaf temperatures were measured in 3 cm from the irritated zone before the local action of heating and blue light with using the Testo 885 thermal imager. Statistical analysis providing these ΔNPQ was described in the Section 3.1. Green shadings show time intervals with significant differences between experimental and control values (*p*<0.05, Student’s test). *, the leaf temperature was significantly differed from the temperature under 456 µmol m^-2^s^-1^ actinic light intensity.

Potentially, the influence of the actinic light intensity on changes in Ф_PSII_ and NPQ induced by the local action of heating and blue light could be caused by different leaf temperatures because the intensive actinic light could heat wheat leaves. **Figure 5A** shows increasing the leaf temperatures (t_leaf_) with increasing the actinic light intensity and, thereby, supports this possibility.

To estimate influence of this factor on photosynthetic changes, we used following statistical analysis. (i) Plants were ranged in accordance with their t_leaf_. This ranking was separately performed for experimental (with local irritations) and control (without irritations) plants and for plants under the 456 and 838 µmol m^-2^s^-1^ actinic light intensities. (ii) Four control and four experimental plants with the lowest t_leaf_ were selected from experiments under the 838 µmol m^-2^s^-1^ actinic light intensity. Average t_leaf_ in this group 1 was 30.7 ± 0.8°C. (iii) Four control and four experimental plants with the lowest t_leaf_ were selected from experiments under the 456 µmol m^-2^s^-1^ actinic light intensity. Average t_leaf_ in this group 2 was 26.3 ± 0.3°C. (iv) Four control and four experimental plants with the highest t_leaf_ were selected from experiments under the 456 µmol m^-2^s^-1^ actinic light intensity. Average t_leaf_ in this group 3 was 29.7 ± 0.3°C. (v) Averaged experimental and control ΔNPQ were calculated for each group.

It should be noted that t_leaf_ were not significantly distinguished in groups 1 (the 838 µmol m^-2^s^-1^ actinic light intensity) and 3 (the 456 µmol m^-2^s^-1^ actinic light intensity); in contrast, t_leaf_ in the group 2 (the 456 µmol m^-2^s^-1^ actinic light intensity) was significantly lower than leaf temperatures in groups 1 and 3. It meant that differences in magnitudes of changes in NPQ between groups 1 and 3 should be related to the actinic light intensity (t_leaf_ were similar, the actinic light intensities were differed); in contrast, that differences in these magnitudes between groups 2 and 3 should be only related to the leaf temperatures (t_leaf_ were differed, the actinic light intensities were same).

Irritation-induced changes in ΔNPQ were analyzed for each group. Only NPQ was analyzed to simplify the investigation. It was shown that the local irritation induced similar NPQ changes in groups 2 and 3 (**Figures 5B, C**); in contrast, these changes were maximal in the group 1 (**Figure 5D**). Therefore, different leaf temperatures were not probable reason of different changes in NPQ induced by local action of the moderate heating and blue light under different intensities of the actinic light.

As a whole, results of the first stage of research were in a good accordance to literature data (Grams et al., 2009; Pavlovič et al., 2011; Yudina et al., 2022), which showed decrease of Ф_PSII_ and increase of NPQ in non-irritated zones of plant after local action of stressors; however, these photosynthetic changes were considered to be caused propagation of DESs (action potentials or variation potentials). In contrast, our previous work (Yudina et al., 2023) showed that HESs were rather observed under using the local action of combination of the moderate heating and blue light.

### 3.2 Local action of combination of heating and blue light on photosynthetic parameters under soil drought

**Figure 6:**
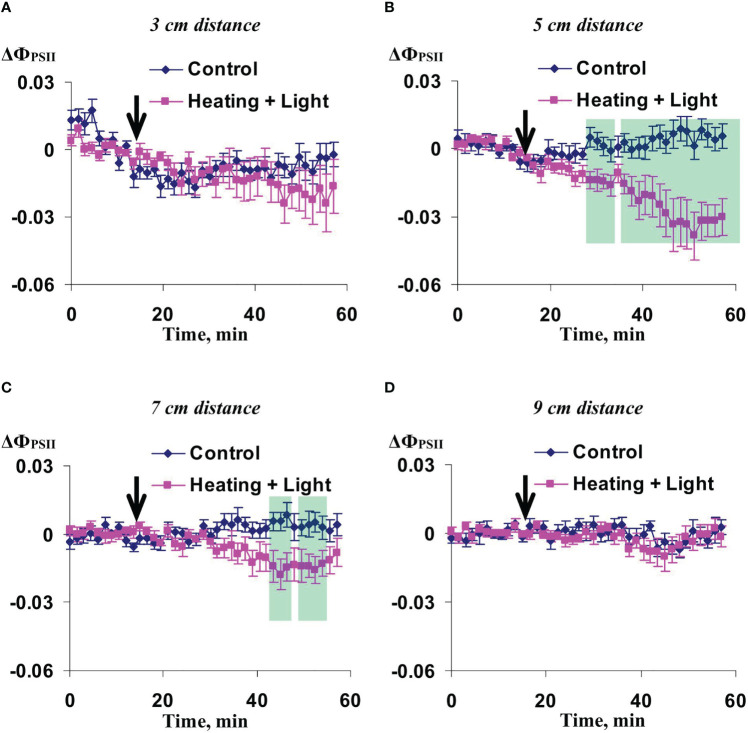
Averaged changes in the quantum yield of photosystem II (ΔФ_PSII_) in 3 cm **(A)**, 5 cm **(B)**, 7 cm **(C)**, and 9 cm **(D)** from zone of the local action of combination of heating and blue light in wheat plants after 7-day soil drought (*n*=12). Arrow marks initiation of this action; control plants were not irritated. ΔФ_PSII_ was calculated as Ф_PSII_ - Ф_PSII_^0^, where Ф_PSII_^0^ was measured before the initiation of the irritation. Intensity of the white actinic light was 456 µmol m^-2^s^-1^. Green shadings show time intervals with significant differences between experimental and control values (*p*<0.05, Student’s test).

Further, we investigated influence of the moderate (7 day) and strong (14 day) soil drought on changes in photosynthetic parameters caused by the local action of combination of heating and blue light. It was shown (**Figure 6**) that the used irritation decreased Ф_PSII_ (value of difference between ΔФ_PSII_ in irritated and control wheat plants was significant and negative) in 5 and 7 cm from the zone of action of heating and blue light under the moderate soil drought. Maximal magnitude and duration of changes was obscured at the 5 cm distance; the large value of difference between ΔФ_PSII_ in irritated and control plants was shown up to termination of the experiment. In contrast, changes in Ф_PSII_ were not significant in 3 and 9 cm from the irritated zone.

**Figure 7:**
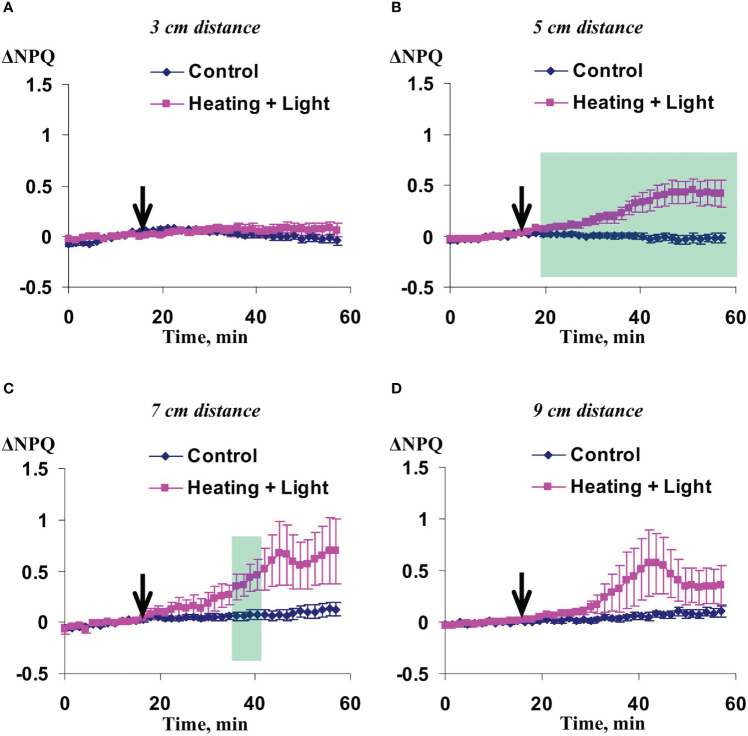
Averaged changes in the non-photochemical quenching of chlorophyll (ΔNPQ) in 3 cm **(A)**, 5 cm **(B)**, 7 cm **(C)**, and 9 cm **(D)** from zone of the local action of combination of heating and blue light in wheat plants after 7-day soil drought (*n*=12). Arrow marks initiation of this action; control plants were not irritated. ΔNPQ was calculated as NPQ - NPQ^0^, where NPQ^0^ was measured before the initiation of the irritation. Intensity of the white actinic light was 456 µmol m^-2^s^-1^. Green shadings show time intervals with significant differences between experimental and control values (*p*<0.05, Student’s test).

Influence of the moderate soil drought on changes in NPQ induced by the irritation was similar with this influence on the changes in the quantum yield of photosystem II (**Figure 7**). The changes were maximal in 5 cm from the irritated zone, intermediate and mainly insignificant in 7 and 9 cm, and fully absent in 3 cm from this zone. It should be noted that changes in NPQ under the moderate soil drought (about 0.5) were strongly higher than ones under the irrigation (about 0.1).

**Figure 8:**
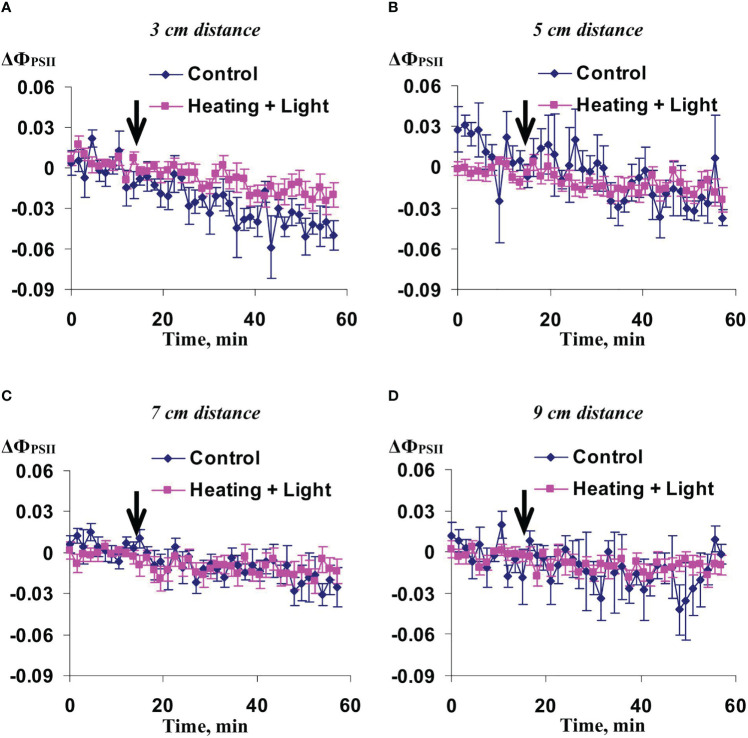
Averaged changes in the quantum yield of photosystem II (ΔФ_PSII_) in 3 cm **(A)**, 5 cm **(B)**, 7 cm **(C)**, and 9 cm **(D)** from zone of the local action of combination of heating and blue light in wheat plants after 14-day soil drought (*n*=12). Arrow marks initiation of this action; control plants were not irritated. ΔФ_PSII_ was calculated as Ф_PSII_ - Ф_PSII_^0^, where Ф_PSII_^0^ was measured before the initiation of the irritation. Intensity of the white actinic light was 456 µmol m^-2^s^-1^.

**Figure 9:**
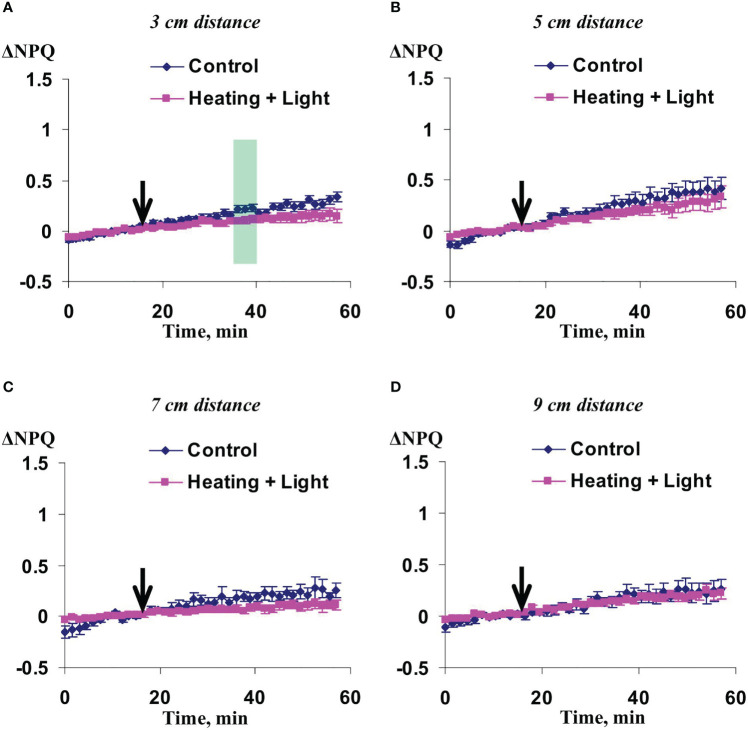
Averaged changes in the non-photochemical quenching of chlorophyll (ΔNPQ) in 3 cm **(A)**, 5 cm **(B)**, 7 cm **(C)**, and 9 cm **(D)** from zone of the local action of combination of heating and blue light in wheat plants after 14-day soil drought (*n*=12). Arrow marks initiation of this action; control plants were not irritated. ΔNPQ was calculated as NPQ - NPQ^0^, where NPQ^0^ was measured before the initiation of the irritation. Intensity of the white actinic light was 456 µmol m^-2^s^-1^. Green shading shows time interval with significant differences between experimental and control values (*p*<0.05, Student’s test).

In contrast, irritation-induced changes in Ф_PSII_ (**Figure 8**) and NPQ (**Figure 9**) were mainly absent in plants deprived of water for 14 days (strong drought treatment); weak significant increase of NPQ (36-39 min after initiation of the local irritation) was only shown. These results were in a good accordance with our previous works which showed suppression of DESs-induced changes in photosynthetic light reactions (Yudina et al., 2022) and decrease of amplitude of HESs (Yudina et al., 2023) under the strong soil drought.

### 3.3 Local action of moderate heating only and blue light only on photosynthetic parameters under irrigated conditions

**Figure 10:**
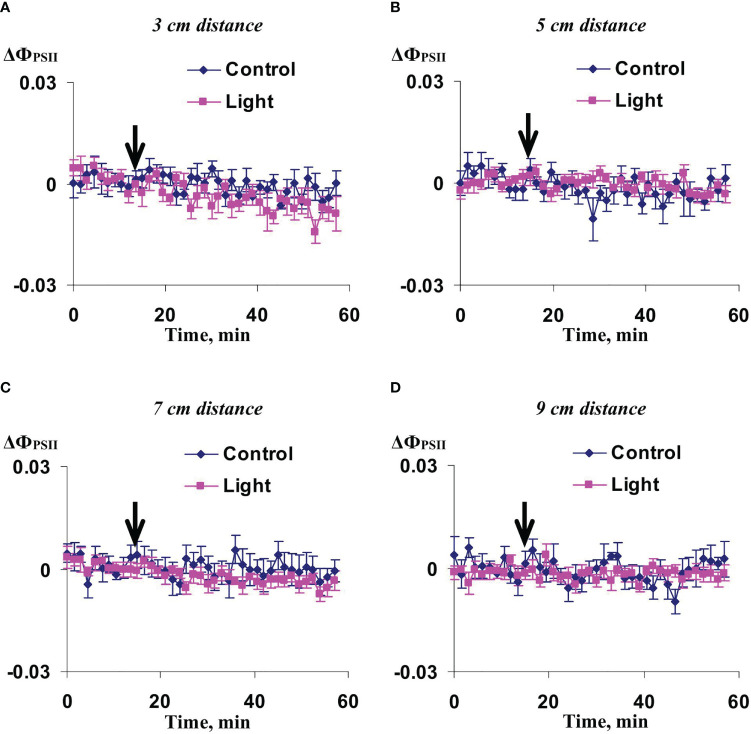
Averaged changes in the quantum yield of photosystem II (ΔФ_PSII_) in 3 cm **(A)**, 5 cm **(B)**, 7 cm **(C)**, and 9 cm **(D)** from zone of the local action of blue light in irrigated wheat plants (*n*=8). Arrow marks initiation of this action; control plants were not irritated. ΔФ_PSII_ was calculated as Ф_PSII_ - Ф_PSII_^0^, where Ф_PSII_^0^ was measured before the initiation of the irritation. Intensity of the white actinic light was 456 µmol m^-2^s^-1^.

**Figure 11:**
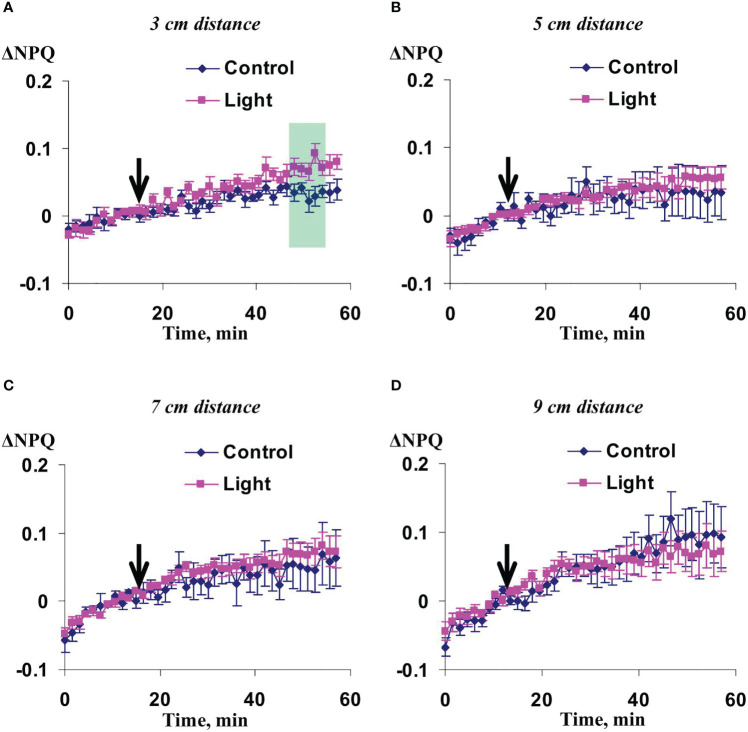
Averaged changes in the non-photochemical quenching of chlorophyll (ΔNPQ) in 3 cm **(A)**, 5 cm **(B)**, 7 cm **(C)**, and 9 cm **(D)** from zone of the local action of blue light in irrigated wheat plants (*n*=8). Arrow marks initiation of this action; control plants were not irritated. ΔNPQ was calculated as NPQ - NPQ^0^, where NPQ^0^ was measured before the initiation of the irritation. Intensity of the white actinic light was 456 µmol m^-2^s^-1^. Green shading shows time interval with significant differences between experimental and control values (*p*<0.05, Student’s test).

**Figure 12:**
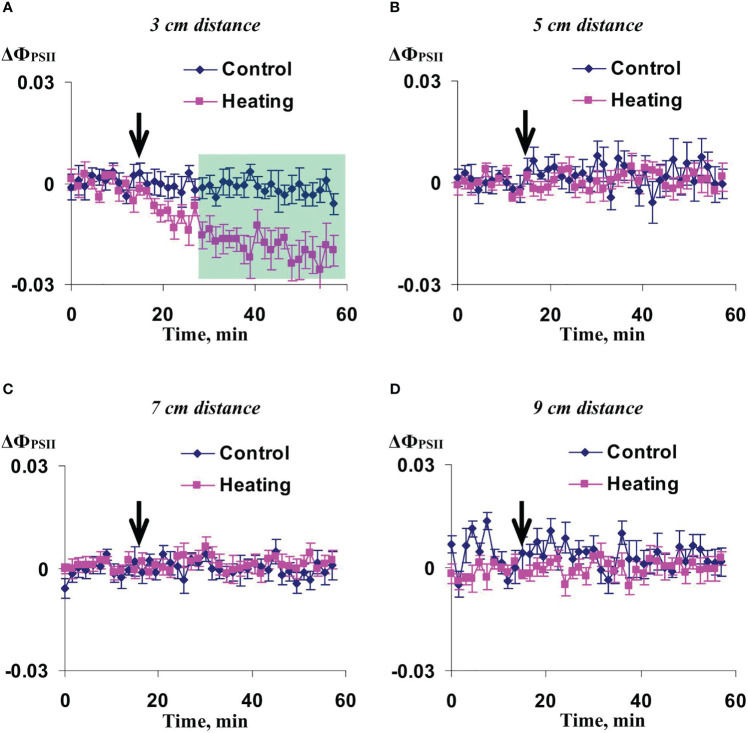
Averaged changes in the quantum yield of photosystem II (ΔФ_PSII_) in 3 cm **(A)**, 5 cm **(B)**, 7 cm **(C)**, and 9 cm **(D)** from zone of the local action of heating in irrigated wheat plants (*n*=8). Arrow marks initiation of this action; control plants were not irritated. ΔФ_PSII_ was calculated as Ф_PSII_ - Ф_PSII_^0^, where Ф_PSII_^0^ was measured before the initiation of the irritation. Intensity of the white actinic light was 456 µmol m^-2^s^-1^. Green shading shows time interval with significant differences between experimental and control values (*p*<0.05, Student’s test).

**Figure 13:**
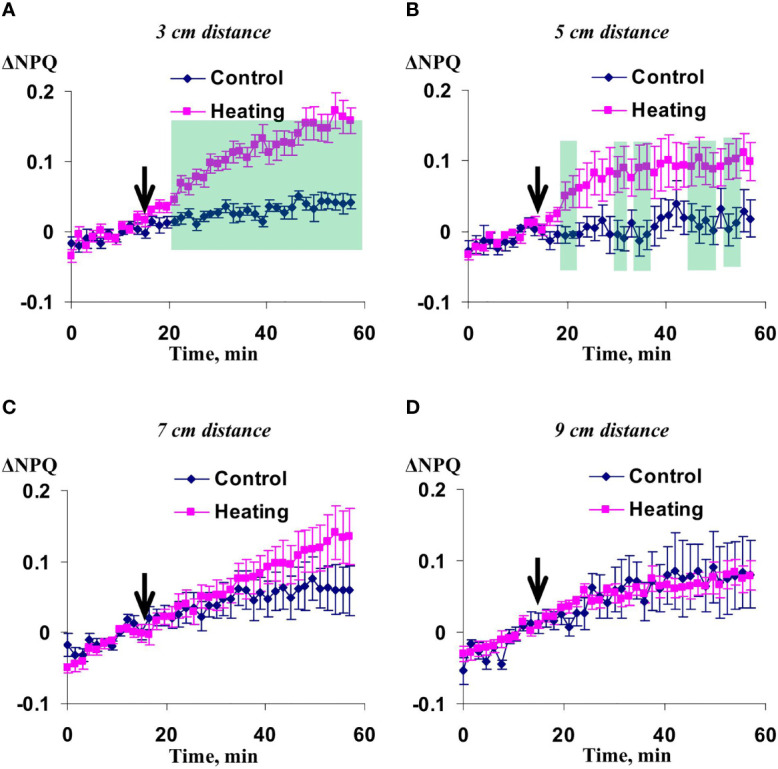
Averaged changes in the non-photochemical quenching of chlorophyll (ΔNPQ) in 3 cm **(A)**, 5 cm **(B)**, 7 cm **(C)**, and 9 cm **(D)** from zone of the local action of heating in irrigated wheat plants (*n*=8). Arrow marks initiation of this action; control plants were not irritated. ΔNPQ was calculated as NPQ - NPQ^0^, where NPQ^0^ was measured before the initiation of the irritation. Intensity of the white actinic light was 456 µmol m^-2^s^-1^. Green shadings show time intervals with significant differences between experimental and control values (*p*<0.05, Student’s test).

Investigation of influence of the local action of the blue light only on Ф_PSII_ and NPQ in non-irritated parts of wheat leaves showed (**Figures 10**, **11**) that both the quantum yield of photosystem II and non-photochemical quenching were not changed after this irritation excluding weak significant increase of NPQ (48-54 min after initiation of the local irritation). In contrast, the local action of the moderate heating only induced the strong decrease of Ф_PSII_ in 3 cm from the irritated zone (**Figure 12A**) and increase of NPQ in 3 cm (**Figure 13A**) and 5 cm (**Figure 13B**). Influence of the local heating on Ф_PSII_ in 5 cm (**Figure 12B**), 7 cm (**Figure 12C**), and 9 cm (**Figure 12D**) from the irritated zone or on NPQ in 7 cm (**Figure 13C**) and 9 cm (**Figure 13D**) was absent.

These results showed that influence of the moderate heating only was similar with the influence of combination of heating and blue light (**Figures 2**, **3**); however, magnitudes of changes in Ф_PSII_ and NPQ induced by the local heating were insignificantly more than ones of similar changes induced by combination of heating and blue light.

As a whole, it meant that just the moderate heating was main mechanism of induction of photosynthetic changes that was in a good accordance with our previous results showing that the moderate heating played a key role in induction of HESs (Yudina et al., 2023) because only weak hyperpolarization signals were caused by the local action of the blue light.

### 3.4 Hyperpolarization signals induced by investigated irritations under irrigated conditions and under soil drought

Finally, we analyzed induction of HESs in different experimental variants, which were used for photosynthetic investigations. These experiments were in accordance with our previous work (Yudina et al., 2023); however, hyperpolarization electrical signals were only investigated in 5 cm from the irritated zone because irritation-induced photosynthetic changes were mainly localized within this distance.

**Figure 14:**
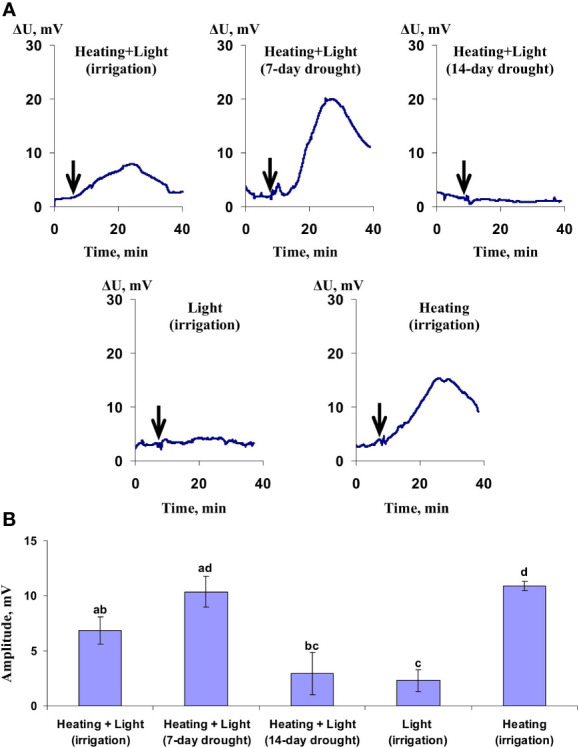
Records of hyperpolarization signals induced by different variants of local irritations (Heating+Light, Light, or Heating) under irrigation, 7-day, or 14-day soil drought **(A)** and average amplitudes of these signals (*n*=7-12) **(B)**. Hyperpolarization signals in the 5 cm distance from the irritated zone were only measured and analyzed. Different letters mark significant differences (*p*<0.05, Student’s test).

It was shown (**Figure 14**) that HESs (which could be identified as system potentials, Zimmermann et al., 2009; Zimmermann et al., 2016) had maximal amplitudes under the local action of combination of the moderate heating and blue light in wheat plants under the 7-day soil drought and under the local action of the moderate heating in irrigated plants. In contrast, these hyperpolarization signals were weak under the local action of light and under the local action of heating and blue light in plants under the 14-day soil drought. HESs induced by combination of the moderate heating and blue light in irrigated wheat plants were intermediate. These results were in an accordance with photosynthetic changes in corresponding experimental variants and supported participation of hyperpolarization electrical signals in induction of changes in photosynthetic light reaction (the decrease of Ф_PSII_ and increase of NPQ). They also showed that just the moderate heating induced these HESs because the local action of the blue light induced weak hyperpolarization signals only. It should be noted that amplitude of the moderate heating-induced HESs was significantly larger than this amplitude of HESs induced by the combined action of the moderate heating and blue light (**Figure 14B**).

## 4 Discussion

Potentially, ESs, which influence numerous physiological processes and modify the plant tolerance to adverse factors (Fromm and Lautner, 2007; van Bel et al., 2014; Gallé et al., 2015; Choi et al., 2016; Hedrich et al., 2016; Szechyńska-Hebda et al., 2017; Farmer et al., 2020; Szechyńska-Hebda et al., 2022), can be the important mechanism of the fast systemic adaptation response of higher plants under the local action of stressors (Sukhov et al., 2019; Sukhova and Sukhov, 2021). However, propagation of action potential, which can be induced by weak and moderate irritations, requires the long-term rest period and absence of chronic action of adverse factors (Sukhov et al., 2019). In contrast, variation potential can be induced under chronic action of moderate stressors (e.g., the moderate soil drought, Yudina et al., 2022); however, strong damages (particularly, burning, heating to 55°C and more, or mechanical wounding) are necessary for its induction (Sukhov et al., 2019; Sukhova and Sukhov, 2021). It means that propagation of action potential and variation potential under environmental conditions cannot be the common phenomenon. Action potential is rather the additional long-distance signal which regulates physiological processes in plants under stable and favorable conditions (Sukhov et al., 2019). Variation potential seems to be the “emergency” long-distance signal which is activated by rare extreme events (e.g., plant burning under wildfire or mechanical wounding under hail) and supports plant survival under these conditions (probably, through sacrificing some cells or organs in plants, Sukhova and Sukhov, 2021).

Thus, DESs (action potential and variation potential) has limitation in their potential participation in the systemic adaptation response of plants to the local action of “physiological” stressors, which are frequent under environmental conditions (e.g., the moderate heating and excess light). It seems to be possible that HESs can play this role. There are some arguments supporting this possibility. (i) In irrigated wheat plants (Yudina et al., 2023), HESs, which are identified as system potentials, can be induced by the combined local action of the moderate heating (40°C) and blue light or the local action of this moderate heating only. These HESs also propagate under the moderate soil drought, which can be a common event under environmental conditions; the propagation is partially suppressed by the strong soil drought only. (ii) There are works showing influence HESs on photosynthetic processes; e.g., both DESs and HESs can be induced by the local action of flame and inactivate photosynthetic processes (suppression of the CO_2_ assimilation and decrease of Ф_PSII_) in poplar (Lautner et al., 2005). Vuralhan-Eckert et al. (2018) show decrease of the photosynthetic CO_2_ assimilation in maize caused by propagation of HESs induced by strong heating of the leaf tip (about 1000°C). HESs, induced by burning of leaves under the strong drought, can also influence photosynthesis in pea plants (Yudina et al., 2022); however, direction of this influence is opposite (activation of the photosynthetic CO_2_ assimilation). The last result seems to be contradictory because the photosynthetic inactivation is typical plant response to ESs (Sukhov et al., 2019), but this activation can be related to stomate closure in plants under the strong drought (Yudina et al., 2022).

There are three main results of the current work. First, we show that hyperpolarization electrical signals, which are induced by the moderate irritation (mainly, moderate heating to 40°) can influence parameters of photosynthetic light reactions (the quantum yield of photosystem II and non-photochemical quenching of chlorophyll fluorescence) in wheat leaves. The participation of HESs in induction of photosynthetic changes is supported by several points. (i) Photosynthetic changes are slower than changes in the surface potential (see, e.g., **Figures 14A**, **3** and **4**). (ii) HESs with higher amplitudes, which are observed under the moderate soil drought or under the local heating only (**Figure 14B**), are accompanied with maximal magnitudes of changes in parameters of photosynthetic light reactions (**Figures 6**, **7**, **12**, and **13**). (iii) HESs with lower amplitudes, which are observed under the strong soil drought or under the local illumination only (**Figure 14B**), are accompanied with low magnitudes of changes in parameters of photosynthetic light reactions (**Figures 7**–**10**).

These results are in a qualitative accordance with literature data (Lautner et al., 2005; Grams et al., 2009) and with our previous results (see, e.g., reviews by Sukhov et al. (2019) and Sukhova and Sukhov (2021)), which show that propagation of DESs is necessary for induction of photosynthetic responses, time of initiation of these responses are dependent on time of propagation of ESs through leaf, and magnitudes of the photosynthetic responses are related to amplitudes of electrical signals. However, revealing relations between photosynthetic changes and HESs is new and important result supporting participation of the hyperpolarization electrical signals in induction of the systemic adaptation response in higher plants.

It should be also noted that there are other methods of analysis of participation of electrical signals in induction of physiological changes including application of local blocks by chilling or by metabolic inhibitor treatment or cutting off the irritated zone of plants in different time intervals after this irritation (Filek and Kościelniak, 1997). The last method was effectively used in investigation of mechanisms of variation potentials in wheat plants (Vodeneev et al., 2012). Considering potential similarity between mechanisms of variation potentials and HESs (Yudina et al., 2022; Yudina et al., 2023), we suppose that this approach can be also used in future analysis of the HESs participation in induction of physiological changes in plants.

Second, our results shows that HESs suppresses photosynthetic light reactions decreasing Ф_PSII_ (**Figures 2**, **4**, **6**, and **12**) and increasing NPQ (**Figures 3**, **4**, **7**, and **13**). This result is in a good accordance with the DESs-induced photosynthetic inactivation including the decrease of the quantum yield of photosystem II (Grams et al., 2009; Pavlovič et al., 2011; Gallé et al., 2013) and increase of the non-photochemical quenching of chlorophyll fluorescence (Pavlovič et al., 2011; Yudina et al., 2022). In accordance with series of our previous works (see reviews by Sukhov (2016); Sukhov et al. (2019) and Sukhova and Sukhov (2021), which are focused on this problem), the DESs-induced photosynthetic inactivation plays an important role in increasing the plant tolerance to stressors through the change in tolerance of photosynthetic machinery (on basis of suppression of the photosynthetic linear electron flow and activation of NPQ and cyclic electron flow around photosystem I), through the increase of the ATP content in leaves, and through the probable modification of production of reactive oxygen species in chloroplasts. As a result, this inactivation participates in forming the systemic adaptation response in higher plants (Sukhov et al., 2019). It can be hypothesized that the similar HESs-induced photosynthetic inactivation also increases the plant tolerance to stressors and, therefore, can participate in this systemic response. This hypothesis seems to be probable; however, it requires future investigations.

The similarity between DESs-induced and HESs-induced photosynthetic changes poses the question: What is mechanism of these changes? In accordance with literature data and our previous results (see reviews by Sukhov (2016) and Sukhova and Sukhov (2021)), DESs-induced photosynthetic inactivation is caused by the transient inactivation of H^+^-ATPase, increasing pH in the apoplast (which decreases CO_2_ flux from the apoplast to the stroma and, thereby, suppresses the photosynthetic dark and light reactions), and decreasing pH in the cytoplasm, stroma, and lumen (which directly suppress the photosynthetic light reactions and stimulate defense mechanisms including NPQ and the cyclic electron flow around photosystem I). This mechanism is in a good accordance with mechanisms of DESs (action potential and variation potential) which include the transient inactivation of H^+^-ATPase in the plasma membrane (Stahlberg et al., 2006; Sukhov, 2016; Sukhova and Sukhov, 2021). On the other hand, HESs (system potentials) are based on the transient activation of H^+^-ATPase in the plasma membrane (Zimmermann et al., 2009) which may be caused by the propagation of the hydraulic wave with moderate magnitude, the followed activation of mechanosensitive Ca^2+^ channels, and intermediate increase of calcium concentration in the cytoplasm (Yudina et al., 2023). Possibility of positive influence of the increased Ca^2+^ concentration on the H^+^-ATPase activity is supported by previous electrophysiological investigations (Lew, 1989; Grinberg et al., 2022).

Decreasing the apoplastic pH is an expected result of this activation; however, the experimental investigation (Zimmermann et al., 2009) shows increasing the apoplastic pH during the system potential generation. This paradoxical effect can be explained on basis of strong decreasing the apoplastic K^+^ concentration, which is observed during generation of system potential (Zimmermann et al., 2009), because K^+^ and H^+^ competes for negatively charged buffer molecules in the apoplast (Gradmann, 2001). It means that this K^+^ decrease can compensate increasing the total proton concentration (free and buffer-bonded protons) and, thereby, decreases concentration of free protons in the apoplast (and increases its pH). Mechanism of the apoplastic K^+^ decrease can be based on the hyperpolarization of the plasma membrane that stimulates the potassium ion influx through increasing the K^+^ motive force and activating potential-dependent inwardly rectifying K^+^ channels (Sukhova et al., 2022).

The apoplastic pH increase caused by generation of system potential (Zimmermann et al., 2009) is very important to explain mechanisms of the HESs-induced photosynthetic inactivation (our current results) because the apoplastic alkalization decreases the CO_2_ mesophyll conductance and suppresses the photosynthetic dark reactions. In accordance with Gallé et al. (2013), decreasing the CO_2_ mesophyll conductance can be caused by pH-dependent changes in aquaporin conductance because they can transfer CO_2_. The alternative mechanism can be based on pH-dependent shifts between CO_2_ and HCO_3_^-^ (Sukhov, 2016) because HCO_3_^-^ is weakly transferred across biological membranes (Tholen and Zhu, 2011); however, our recent model-based analysis showed that these shifts weakly influence the photosynthetic CO_2_ assimilation (Sukhova et al., 2022).

The inactivation of the photosynthetic dark reactions suppresses the photosynthetic light reactions (Sukhov, 2016). This suppression is based on increasing ATP : ADP and NADPH : NADP^+^ ratios in the stroma of chloroplasts (their consumption in the photosynthetic dark reactions is low) and following decreasing activity of H^+^-ATPsynthase and increasing the proton gradient across the thylakoid membrane (Pavlovič et al., 2011; Sukhov, 2016). This increased proton gradient suppresses the photosynthetic linear electron flow (Tikhonov, 2013) (i.e., decreases Ф_PSII_) and stimulates NPQ (Ruban, 2016).

Increasing magnitudes of HESs-induced photosynthetic changes with increasing intensity of the actinic light is in a good accordance with this hypothetical mechanism because (i) increasing the actinic light intensity stimulates the photosynthetic linear and cyclic electron flows (see, e.g., Zivcak et al., 2013) and (ii) mismatch between the intensive linear electron flow and suppressed CO_2_ assimilation, caused by the decreased CO_2_ mesophyll conductance (Gallé et al., 2013), is the main reason of electrical signals-induced changes in parameters of photosynthetic light reactions (Pavlovič et al., 2011; Sukhov, 2016). It means that the light-dependent increase of the linear electron flow (which provides the proton gradient across the thylakoid membrane) before irritation should increases magnitude of changes in this gradient after the ESs-induced suppression of photosynthetic dark reactions; in turn, these changes stimulate increasing NPQ (Ruban, 2016) and decreasing Ф_PSII_ (Tikhonov, 2013).

Third, our work shows that the moderate soil drought can increase magnitude of changes in Ф_PSII_ and NPQ (**Figures 6**, **7**) and shifts a region of the photosynthetic changes on larger distance from the irritated zone. In contrast, the strong soil drought fully suppresses these changes in wheat plants (**Figures 8**, **9**). These results are in a good accordance with suppression by the strong water deficit of changes in photosynthetic light reactions caused by burning-induced DESs in pea plants (Yudina et al., 2022) and show possibility of regulation of the HESs-induced photosynthetic response by environmental conditions. It can be proposed that increasing magnitudes of the photosynthetic changes under the moderate soil drought is related to necessity of strong activation of photosynthetic defense mechanisms under adverse conditions (“usual” changes can be ineffective in stressed plants). In contrast, the additional activation of photosynthetic defense mechanisms can be rather harmful under the strong soil drought (Sukhov et al., 2019; Sukhova and Sukhov, 2021) because these mechanisms are already activated under the strongly adverse conditions.

It is probably that suppression of propagation of HESs (**Figure 14**) is main mechanism of weak photosynthetic changes under the strong soil drought (**Figures 8**, **9**). Early we hypothesized (Yudina et al., 2022; Yudina et al., 2023) that the strong soil drought decreases the water content in plants and, therefore, decrease a water flux from cells to xylem vessels under the irritation (e.g., the moderate heating). Decreasing this water flux lowers increasing the hydraulic pressure in the irritated zone and, thereby, weakens propagation of the hydraulic wave in plants. As a result, propagation of HESs can be suppressed under these conditions.

Specific mechanisms of the positive influence of the moderate soil drought on magnitudes of photosynthetic changes (**Figures 6**, **7**) are not fully clear, now. Partially, it can be caused by increasing amplitude of HESs (**Figure 14B**); however, this increase is not significant. Our previous model-based analysis (Sukhova et al., 2022) showed that influence of ESs-induced decreasing the CO_2_ mesophyll conductance on photosynthesis is stimulated with lowering quantity of open stomata. Considering closure of stomata under drought (Zivcak et al., 2013), we suppose that this closure can be potential mechanism of increasing magnitude of HESs-induced photosynthetic changes under the moderate soil drought. On the other hand, mechanisms of the shift of localization of photosynthetic changes under the moderate soil drought seem to require future investigations.

Additionally, magnitude of HESs-induced changes in Ф_PSII_ and NPQ are dependent on the actinic light intensity (**Figure 4**). It shows that light conditions in the non-irritated parts of plants can also modify photosynthetic changes induced by the hyperpolarization signals (potential mechanisms of these modification are discussed above).

Finally, it should be additionally noted that induction of HESs by local action of the moderate heating only and almost complete absence of this induction by local action of blue light (**Figure 14**) shows that the moderate heating is the main inductor of HESs; i.e. HESs are similar to variation potential which is mainly induced by strong heating or burning (Sukhov et al., 2019). This result is in a good accordance with our hypothesis (Yudina et al., 2022; Yudina et al., 2023) about participation of the heating-induced hydraulic signal in induction of both variation potential (under high magnitude of the hydraulic wave) and system potential (under low or moderate magnitude of the hydraulic wave). On the other hand, the local illumination by the blue light induces weak HESs (**Figure 14**) and causes the weak increase of NPQ (**Figure 11A**). It shows that regulation of photosynthetic parameters by light-induced HESs cannot be fully excluded; maybe, it can be more important under using other light conditions or plant species. Works (Szechyńska-Hebda et al., 2010; Szechyńska-Hebda et al., 2017) showing participation of light-induced electrical signals in photosynthetic regulation supports this probability.

**Figure 15:**
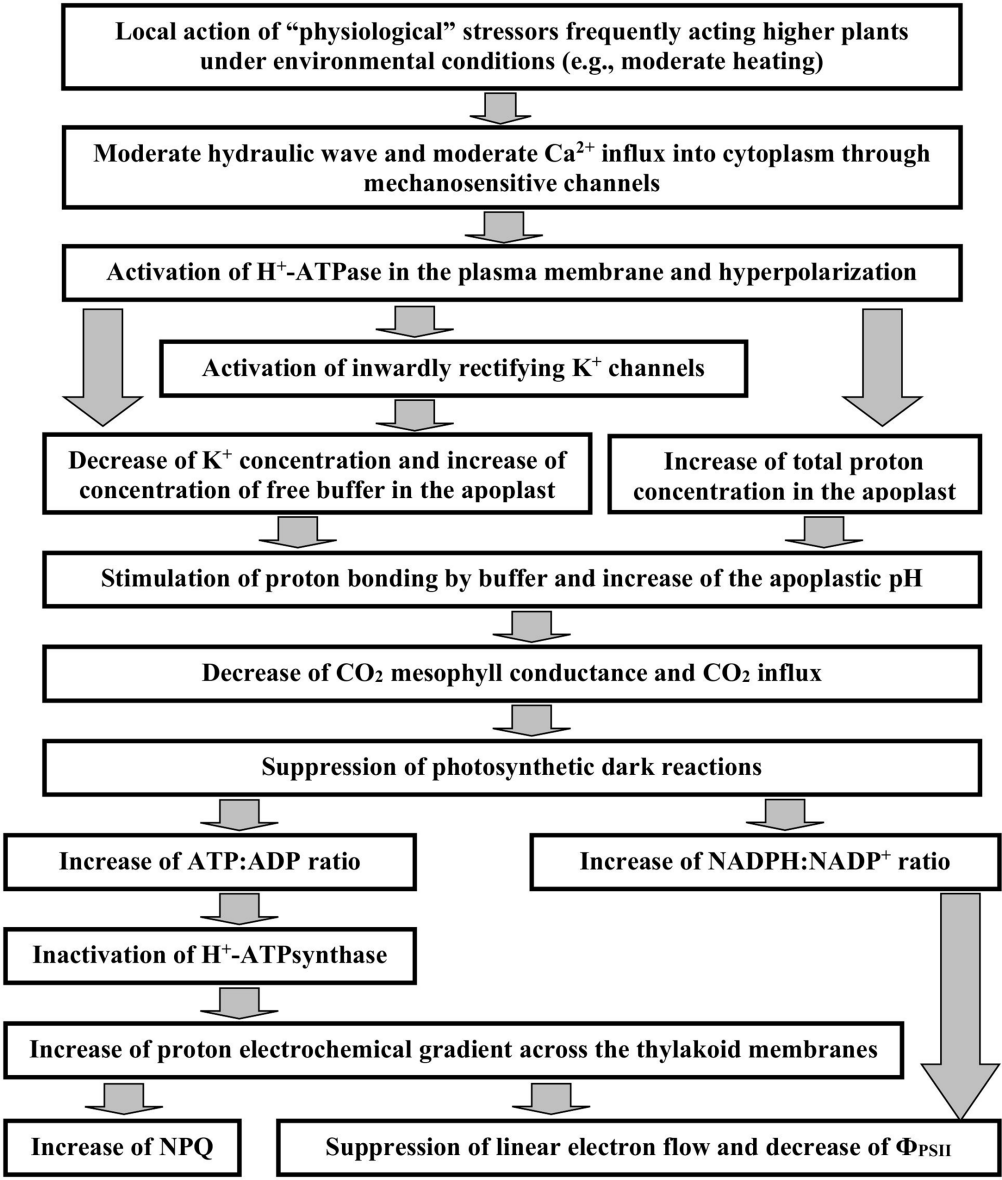
A scheme of hypothetical ways of influence of hyperpolarization electrical signals (system potentials) on photosynthetic processes (see Discussion for details). The scheme is based on results of the current work and our previous results summarizing in reviews (Sukhov, 2016; Sukhov et al., 2019; Sukhova and Sukhov, 2021).

Thus, our work shows that the local action of “physiological” stressors (at least, the moderate heating) induces propagation of HESs in wheat plants. These hyperpolarization signals can inactivate photosynthesis (decreases the quantum yield of photosystem II and increases the non-photochemical quenching of chlorophyll fluorescence) and, probably, participates in the system adaptation response in higher plants. **Figure 15** summarizes hypothetical ways of influence of HESs on photosynthetic processes. Additionally, our results show that the HESs-induced photosynthetic response can be regulated by environmental conditions (the soil drought and actinic light intensity).

As a whole our results reveal participation of HESs (system potentials) in induction of the system photosynthetic response in plants under local action of moderate stressors (particularly, heating). Previously, this role was clearly shown for DESs only (see our reviews Sukhov, 2016; Sukhov et al., 2019); however, their participation in the photosynthetic regulation in higher plants under natural conditions are limited by the necessity of stable and favorable conditions (for action potentials) or necessity of local action of the strong damages (for variation potentials) (Sukhov et al., 2019; Yudina et al., 2023). The HESs participation, probably, excludes these limitations and supports important role of electrical signals in photosynthetic regulation under environmental conditions.

## Data availability statement

The raw data supporting the conclusions of this article will be made available by the authors, without undue reservation.

## Author contributions

Conceptualization, LY, ES, and VS; methodology, LY, AP, YZ, KA, and KG; formal analysis, ES and VS; investigation, LY, AP, YZ, KA, and KG; writing—original draft preparation, LY, ES and VS; writing—review and editing, VS; supervision, VS; project administration, LY; funding acquisition, LY. All authors contributed to the article and approved the submitted version.
